# Properties and Roles of γδT Cells in *Plasmodium yoelii nigeriensis* NSM Infected C57BL/6 Mice

**DOI:** 10.3389/fcimb.2021.788546

**Published:** 2022-01-20

**Authors:** Hongyan Xie, Shihao Xie, Mei Wang, Haixia Wei, He Huang, Anqi Xie, Jiajie Li, Chao Fang, Feihu Shi, Quan Yang, Yanwei Qi, Zhinan Yin, Xinhua Wang, Jun Huang

**Affiliations:** ^1^ State Key Laboratory of Respiratory Disease, Guangzhou Institute of Respiratory Health, The First Affiliated Hospital of Guangzhou Medical University, Guangzhou, China; ^2^ Zhuhai Precision Medical Center, Zhuhai People’s Hospital (Zhuhai Hospital Affiliated with Jinan University), Jinan University, Zhuhai, China; ^3^ The Biomedical Translational Research Institute, Faculty of Medical Science, Jinan University, Guangzhou, China; ^4^ Key Laboratory of Immunology, Sino-French Hoffmann Institute, Guangzhou Medical University, Guangzhou, China

**Keywords:** *Plasmodium yoelii nigeriensis* NSM, γδT cells, single-cell RNA sequencing, T cell, B cell

## Abstract

**Background:**

Many kinds of immune cells are involved in malaria infection. γδT cells represent a special type of immune cell between natural and adaptive immune cells that play critical roles in anti-parasite infection.

**Methods:**

In this study, malaria infection model was constructed. Distribution of γδT cells in various immune organs and dynamic changes of γδT cells in the spleens of C57BL/6 mice after infection were detected by flow cytometry. And activation status of γδT cells was detected by flow cytometry. Then γδT cells in naive and infected mice were sorted and performed single-cell RNA sequencing (scRNA-seq). Finally, γδTCR KO mice model was constructed and the effect of γδT cell depletion on mouse T and B cell immunity against *Plasmodium* infection was explored.

**Results:**

Here, splenic γδT cells were found to increase significantly on day 14 after *Plasmodium yoelii nigeriensis* NSM infection in C57BL/6 mice. Higher level of CD69, ICOS and PD-1, lower level of CD62L, and decreased IFN-γ producing after stimulation by PMA and ionomycin were found in γδT cells from infected mice, compared with naive mice. Moreover, 11 clusters were identified in γδT cells by scRNA-seq based t-SNE analysis. Cluster 4, 5, and 7 in γδT cells from infected mice were found the expression of numerous genes involved in immune response. In the same time, the GO enrichment analysis revealed that the marker genes in the infection group were involved in innate and adaptive immunity, pathway enrichment analysis identified the marker genes in the infected group shared many key signalling molecules with other cells or against pathogen infection. Furthermore, increased parasitaemia, decreased numbers of RBC and PLT, and increased numbers of WBC were found in the peripheral blood from γδTCR KO mice. Finally, lower IFN-γ and CD69 expressing CD4^+^ and CD8^+^ T cells, lower B cell percentage and numbers, and less CD69 expressing B cells were found in the spleen from γδTCR KO infected mice, and lower levels of IgG and IgM antibodies in the serum were also observed than WT mice.

**Conclusions:**

Overall, this study demonstrates the diversity of γδT cells in the spleen of *Plasmodium yoelii nigeriensis* NSM infected C57BL/6 mice at both the protein and RNA levels, and suggests that the expansion of γδT cells in cluster 4, 5 and 7 could promote both cellular and humoral immune responses.

## Introduction

Malaria is one of the largest causes of morbidity and mortality in tropical and subtropical regions of the world (Saavedra-Langer et al., 2018). It is transmitted to humans through the infected anopheles mosquitoes. Human malaria is caused by infected with different *Plasmodium* species, including *Plasmodium falciparum*, *Plasmodium malariae, Plasmodium ovale, Plasmodium vivax*, and *Plasmodium knowlesi* (Ortiz-Ruiz et al., 2018). *P. yoelii nigeriensis* NSM is a subspecies of the rodent malaria parasite that provides an important animal model for studies of malaria pathogenesis (Li et al., 2016). In the experimental *Plasmodium i*nfection model, *Plasmodium* development directly enters erythrocytic cycle. The infected red blood cells (iRBCs) cause damage to multiple organs through the blood circulatory system, such as the spleen, liver, and lung (Wei et al., 2021). However, some of the infected mice could recover without treatment after about, one month later.

The spleen is a major peripheral immune organ that performs critical physiological functions. It serves as a quality control mechanism for removing senescent red blood cells (RBCs), infected red blood cells (iRBCs), and infectious microorganisms in the process of dealing with parasite invasion (Elizalde-Torrent et al., 2021). Differences in the ability of the spleen to deal with iRBCs are linked to differences in *Plasmodium* virulence (Huang et al., 2016). Malaria infection leads to hyper reactive malarial splenomegaly syndrome, and the spleen becomes a primary organ for eliminating iRBCs (White, 2017). *Plasmodium* infection can induce significant responses of splenic T cells (Hirunpetcharat and Good, 1998; Wipasa et al., 2001; Xu et al., 2002). Early responses in the spleen are key factors modulating the clinical outcome of malaria infection (Huang et al., 2016).

Many kinds of immune cells are involved in the processes and mechanisms of the malaria-induced immune response (Abel et al., 2018; Akbari et al., 2018). The patterns of the immune response display essential roles in malaria progression (Chaves et al., 2016; Keswani et al., 2016; Lopez et al., 2017). Specifically, CD4^+^ T-cell responses have been associated with control of erythrocytic stage parasites, but a small number of studies indicate a helper role also in pre-erythrocytic immunity (Perez-Mazliah and Langhorne, 2014). The B cell-mediated humoral immune response could mediate the antimalarial response and even induce memory B cell development (Sundling et al., 2019; Aye et al., 2020).

Although the proportions of γδT cells are less than 5% in both mice and human immune cells (Nielsen et al., 2017), γδ TCR ligands do not generally require processing or presentation by major histocompatibility complex (MHC) antigens (Born et al., 2013). It can function as antigen-presenting cells (APCs) (Tyler et al., 2017), expressing numerous APC-related cell surface markers (Khan et al., 2014). γδT cells can secrete cytokines, such as IFN-γ, IL-4, IL-17, transforming growth factor beta (TGF-β), and granulocyte macrophage colony stimulating factor (GM-CSF), to regulate the migration of other immune cells, bring about lysis of infected cells by secreting granzymes (granzyme A and B), provide help to B cells and induce IgE production, present antigen to conventional T cells, activate antigen presenting cells (APC) maturation, and are also known to produce growth factors that regulate the stromal cell function (Silva-Santos et al., 2019; Cha et al., 2020; Seifert et al., 2020). It is reported that γδT cells play critical roles in the development of asthma (Victor et al., 2020), oral mucosa (Hovav et al., 2020), and tumors immunity (Wu et al., 2017).

γδT cells are a heterogeneous cell population with different subsets playing specialized and often opposing roles during immune responses. Human γδT cells can be divided into three populations based on δ chain expression(Vδ1^+^, Vδ2^+^, and Vδ3^+^ γδT cells). The Vδ2^+^ T cells can be divided into Vγ9^+^Vδ2^+^ and Vγ9^-^Vδ2^+^ subsets (Zhou et al., 2020). The major adult γδT cell subsets are the Vγ1.1^+^ and Vγ2^+^ γδT cells that can be found in both epithelial tissues and secondary lymphoid organs in mice (Carding and Egan, 2002). In response to different cytokines, γδ T cells can shift from one phenotype to another, in a process referred to as polarization. It has been demonstrated that γδT cells can be polarized into γδ T1 cells(producing IFN-γ and TNF-α), γδ T2 cells (producing increased IL-4) and γδ T17 cells (producing only IL-17) depending on the priming cytokine milieu (Caccamo et al., 2011). Moreover, it can polarize towards follicular B-helper T cells (γδ Tfh cells), regulatory γδT cells (γδ Treg cells) following stimulation with different cytokines regulatory γδT cells (γδTreg) (Wu et al., 2017). It was reported that some γδT cells can express CD4 and CD8 molecules on the surface (Yang et al., 2021). Therefore, CD4 and CD8 can also be used to distinguish the subtype of γδT cells. Moreover, IL-17A/F producing Vgamma4^+^ Vdelta4^+^ T cells was found to be a long lasting resident memory T-cell (TRM) population, which persisted in the dermis for long periods of time after initial stimulation with Aldara (Hartwig et al., 2015). CD39^+^ γδT cell was found with tissue-resident memory phenotype which may contribution to the pathogenesis of IBD and other inflammatory disorders (Libera et al., 2020).

γδT cells have long been known to rapidly proliferate following primary malaria infection in humans and mice (Dantzler and Jagannathan, 2018). *P. falciparum* infection in children, malaria-naive adults, and malaria-experienced adults results in the expansion of γδT cells (Hviid et al., 2001). γδT cells can form immunological synapses with and lysis iRBCs (Junqueira et al., 2021), and destroy blood residing *P*. *falciparum* (Hernandez-Castaneda et al., 2020). Mice without γδT cells suppressed and reduce a primary infection of *P. chabaudi* with a slight delay in the time of clearance of the acute phase of infection and significantly higher recrudescent parasitaemia compared with naive control mice (Langhorne and Holder, 1998). *Plasmodium* infection could induce “memory-like imprints” in γδ T cells to promote γδ T cell mediated antigen presentation during subsequent infections (Kumarasingha et al., 2020). Regulated γδT cell responses may be critical to balance immune protection with severe pathology rely on proinflammatory cytokines, such as IFN-γ (Pamplona and Silva-Santos, 2020).

In this study, the properties and roles of γδT cells in the spleen of *Plasmodium yoelii nigeriensis* NSM infected C57BL/6 mice were investigated, and the mechanism was explored.

## Methods

### Mice

6-8 weeks old female SPF C57BL/6 mice were purchased from the medical laboratory animal center of Guangzhou University of Chinese Medicine and the γδTCR knockout (KO) mice (B6.129P2-Tcrd^tm1Mom^/J, 002120) were provided by Jinan University (Sun et al., 2018). All animal experiments were performed in strictly accordance with the Regulations for the Administration of Affairs Concerning Experimental Animals (1988.11.1). All protocols for animal use were approved to be appropriate and humane by the institutional animal care and use committee of Guangzhou Medical University (2015–012). Every effort was made to minimize suffering.

### Parasites and Infection

*Plasmodium yoelii nigeriensis* NSM was purchased from the malaria research and reference reagent resource center (MR4). The frozen *Plasmodium yoelii* was removed from the liquid nitrogen tank, followed by 37°C water bath thawing and resuscitate after 1 min, and placed on the ice. After intra-peritoneal injection of C57BL/6 mice with *Plasmodium yoelii* (200 ul/mice), blood was collected through tail vein and diluted in 1:1000 proportion to sterile PBS solution when the parasitaemia up to 10%-15% after 2-3 days. 6-8 weeks female C57BL/6 mice were divided into two groups (infection group and control group). 1×10^6^ infected red blood cells (iRBC) were injected into the infection group C57BL/6 mice through tail vein. 24h after infection, the blood was obtained from the tail tip of mice to prepare blood film. After being fixed by methanol and stained with Giemsa, the parasitaemia was examined by optical microscopy. And the changes of parasitaemia were monitored in WT-mice and γδTKO-mice every day. In addition the survival rate of the mice was calculated.

### Antibodies

BV-510 conjugated anti-mouse CD3 (145-2C11), PerCP-Cy5.5 conjugated anti-mouse CD4 (RM4-5), APC-cy7 conjugated anti-mouse CD8 (53-6.7), FITC conjugated anti-mouse γδTCR (GL3), PE-CD25 conjugated anti-mouse (A7R34), APC-conjugated anti-mouse CD69 (H1.2F3), APC-conjugated anti-mouse CD44 (IM7), PE-cy7 conjugated anti-mouse CD278 (ICOS, 15F9), Percp5.5 conjugated anti-mouse CD16/32 (93), APC conjugated anti-mouse IFN-γ (XMG1.2), PE conjugated anti-mouse IL-4 (11B11), PE conjugated anti-mouse IL-17 (TC11-18H10), PE conjugated anti-mouse IL-10 (JES5-16E3), APC conjugated anti-mouse IL-5(TRFK5), PE conjugated anti-mouse IL-2 (561061), PE conjugated anti-mouse CD154 (MR1), PE conjugated anti-mouse CD183 (CXCR3-173), PE conjugated anti-mouse CD80 (16-10A1), matched control monoclonal antibodies (MG1-45) were purchased from BioLegend (San Diego, CA, USA).

### Lymphocyte Isolation

Mice were sacrificed at different time points after malaria infection. The liver, lung, blood, spleen, and peripheral blood mononuclear cell (PBMC) were collected, firstly. Then lung was cut to small pieces and incubated in 5 ml of digestion buffer (collagenase IV/DNase I mix, Invitrogen Corporation) for 30 min at 37 °C. The digested lung tissue was pressed through 200-gauge stainless-steel mesh, and then was suspended in Hank’s balanced salt solution (HBSS). Liver, lung, spleen, and mesenteric lymph nodes (MLN) were mechanically dissociated and processed through a 100-μm cell strainer (BD Falcon), and suspended in HBSS. Lymphocytes were isolated by Ficoll-Hypaque (DAKEWE) density gradient centrifugation. Isolated cells were washed twice in HBSS and re-suspended at 2×10^6^ cells/ml in complete RPMI 1640 medium supplemented with 10% heat-inactivated fetal calf serum (FCS), 100 U/ml penicillin, 100 μg/ml streptomycin, 2 mM glutamine, and 50 μM 2-mercaptoethanol.

### Cell Surface Staining

Cells were washed twice with PBS and blocked in PBS buffer containing 1% BSA for 30 min. Cells were then stained for 30 min at 4 °C in the dark with conjugated antibodies specific for the cell surface antigens CD3, CD4, CD8, γδ T, CD25, CD44, CD69,Vγ2, CD62L, CD40L, CD16/32, and PD-1. Cells were analyzed using a flow cytometer (Beckman CytoFLEX), and the results were analyzed using CytExpert 1.1 software (Beckman Coulter, Inc.). Isotype-matched controls for cytokines were included in each staining protocol.

### Intracellular Cytokines Staining

Single lymphocyte suspensions were isolated from the spleen of control and infected mice, and the cell concentration was adjusted to 2×10^6^/ml. Cells were then stimulated with phorbol 12-myristate 13-acetate (PMA) (20 ng/ml, Sigma) and ionomycin (1 μg/ml, Sigma) for 5 h (37°C, 5% CO_2_). Brefeldin A (BFA, 10 μg/ml, Sigma) was added during the last 4 h of incubation. Cells were washed twice in PBS and then stained for 30 min at 4°Cin the dark with conjugated antibodies specific for the cell surface antigens CD3 and γδTCR. Cells were fixed by 4% paraformaldehyde and permeabilized overnight at 4°Cin PBS buffer containing 0.1% saponin (Sigma), 1% BSA and 0.05% NaN_3_. Next, cells were stained with different fluorescence conjugated antibodies specific for cytokines IL-4, IFN-γ, IL-17, IL-2, IL-10, and IL-5. Cells were analyzed using a flow cytometer (Beckman CytoFLEX) and the results were analyzed using CytExpert 1.1 software (Beckman Coulter, Inc.). Isotype-matched controls for cytokines were included in each staining protocol.

### 10× Genomics Chromium Analysis

Spleens were obtained from three naive and three infected mice on day 14 post-infection. Due to the low frequency of γδT cells in mouse spleen, we mixed the splenocytes of three mice in the same group. Single cell solution was prepared and CD3^+^γδTCR^+^ cells were sorted by FACS (Beckman MoFlo). The viability of γδT cells exceed 90% (hoechst H33342/PI staining). Cells were collected, and the expression of RNA in each cell were detected by 10× Genomics Chromium Single Cell RNA Sequencing (See et al., 2018) by LC biotechnology (LTD, Hangzhou, China). In brief, GemCode™ Single Cell platform (10× Genomics, Pleasanton, CA) was used to determine the transcriptomes of single cells. The Chromium Single Cell 3′Library & Gel Bead Kit v3 (10×Genomics, 1000075) was used for single-cell barcoding, cDNA synthesis and library preparation. Libraries were sequenced on Illumina Nova seq6000 using paired-end 150 bp.

The subsequently scRNA-seq data processing and analysis was also done by LC biotechnology. In brief, Seurat implements a graph-based clustering approach. Distances between the cells are calculated based on previously identified PCs. Seurat approach was heavily inspired by recent manuscripts which applied graph-based clustering approaches to scRNA-seq data – SNN-Cliq (Xu and Su, 2015) and CyTOF data-PhenoGraph (Levine et al., 2015). To cluster the cells, modularity optimization techniques -SLM (Subelj and Bajec, 2011) were applied to iteratively group cells together, with the goal of optimizing the standard modularity function.

CellRanger (version 3.1.0) was used, aligned reads on the GRCm38 reference genome for mouse and generated unique molecular identifier gene expression profiles for every single cell under standard sequencing quality threshold (default parameters). Low-quality cells were removed for downstream analysis when they met the following criteria for retaining cells: (1) ≥50,000 sequence reads; (2) ≥40% of reads uniquely aligned to the genome; (3) ≥40% of these reads mapping to RefSeq annotated exons. Through Seurat (Version 3.6.0) R package, we processed the UMI counts mentioned above with further filteration criteria (cells are removed): 1) less than 500 and more than 4000 expressed genes, 2) higher than 10% mitochondrial genome transcript, 3) Genes expressed in less than 3 cells, 4) more than 8000 UMI counts. In total, 3022 cells in infected group and 6109 cells in normal group were captured and sequenced, and 27998 genes were analysed.

Differentially expressed genes (DEGs) were identified by “FindMarkers” function in Seurat using “wilcox” test methods and Bonferroni correction. Significant DEGs were selected from genes with *P* ≤ 0.01 and log processed average fold change (avg_log2FC) ≥ 0.36 for further analysis and visualization. GO analysis and KEGG pathway enrichment analysis for these significant DEGs were performed by clusterProfiler package.

### Enzyme Linked Immunosorbent Assay (ELISA)

Immunoglobulin (Ig) G and IgM antibodies to malaria were measured by ELISA. Briefly, 13-mer peptide with a sequence NH2-SCKNEWGWSKSCS-COOH (Dutta et al., 2018) was synthesized by Ang tuolai biotechnology co. LTD (Zhejiang, China). The peptide was diluted in 0.05 M sodium bicarbonate contained coating buffer (pH 9.6), 10 μg/ml (100 μl/well), at 4°C overnight. The plate was washed twice, and blocked at 4°C for 1 hr. After washing for three times, 100 μl of 100 fold diluted serum was added to each well, and incubated at 37°C for 2 hr. After five times washes, 100 μl horseradish peroxidise (HRP)-conjugated goat anti-mouse IgG (ZB2305, ZSGB-Bio, Beijing, China) and HRP-conjugated goat anti-mouse IgM (RS030210; ImmunoWay Biotechnology, Plano, TX, USA) diluted in PBS/Tween-20 was added and incubated at 37°C for 1h. The plate was washed five times, TMB Substrate Reagent (555214, BD) (100 μl per well) was added and incubated for 10 min in the dark. The reaction was stopped by stop solution and the absorbance of each well was measured at 450 nm with an ELISA plate reader (Model ELX-800; BioTek).

### Blood Cell Analysis

Blood was collected from mice by using a retro-orbital puncture. The numbers of Red blood cell (RBC), white blood cell (WBC), and Platelet (PLT) in the blood were detected and analyzed by an automatic cellular analyzers (DXH-800, Beckman Coulter) (Bigorra et al., 2019).

### Statistical Analysis

Data were analyzed with SPSS 11.0 software (SPSS Inc., Chicago, IL, USA) and GraphPad Prism (v8.02). Differences between the two groups were analyzed in GraphPad Prism (v8.02) using an unpaired t-tests with equal variance and normal distributions. To compare more than two groups, one-way ANOVA and LSD test by SPSS software package and SPSS software were used with equal variance and normal distributions. In addition, Mann-Whitney U test was used with unequal variance or abnormal distributions. The statistical significance was defined as *P* < 0.05.

## Results

### Changes of γδT Cells in Different Organs of C57BL/6 Mice After *P. yoelii* NSM Infection

**Figure 1:**
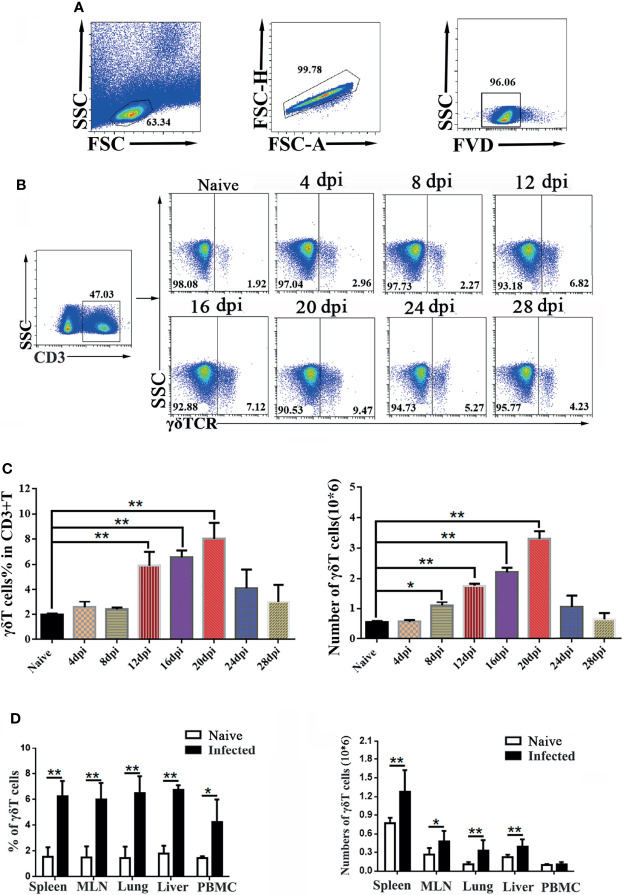
Dynamic changes in γδT cells in the spleen of *P. yoelii* NSM infected mice. Fourteen days after *Plasmodium* infection, lymphocytes from the spleen, MLN, lung, liver, and PBMC of C57BL/6 mice were isolated. Different fluorescence-labelled mAbs against mouse CD3 and γδTCR were used to detect the content of γδT cells by flow cytometry. **(A)** All the doublet cells, dead cells, and nonlymphoid cells were excluded from flow cytometry data. **(B, C)** Dynamic changes in γδT cells in the spleens of C57BL/6 mice infected with *Plasmodium* were determined by FCM from day 4 to day 28, simultaneously setting up naive control. **(D)** Averages of γδT cells in infected mice in different organs were calculated after FCM analysis. Cell numbers of γδT cells from different organs in infected mice, compared with naive groups. Representative results of three independent results are shown. The average of three independent experiments with 3-5 mice per group was shown and repeated three times with similar results. The error bars are SD, *P < 0.05, **P < 0.01.

To explore the role of γδT cells in C57BL/6 mice, dynamic changes in the proportions of γδT cells in the spleen of mice were detected by FCM at days 0, 4, 8, 12, 16, 20, 24 and 28 after *P. yoelii* NSM infection (**Figures 1A, B**). As shown in **Figure 1C**, the results indicated that the percentage of CD3^+^γδTCR^+^ cells in CD3^+^T cells in the spleens of naive mice was 2.1 ± 0.18%. The percentage of CD3^+^γδTCR^+^ cells increased slightly from day 4 to day 8, but significantly increased at days 12, 16 and 20 (*P* < 0.01), and then decreased at days 24 and 28. However, the numbers of detected splenic CD3^+^ γδTCR^+^ cells increased from day 8, peaked at day 20, and then decreased from day 20 to day 28 (**Figure 1C**). Therefore, day 14 was selected as the time point to detect the properties of splenic γδT cells in this study.

To explore the alteration of γδT cells in different organs, C57BL/6 mice were infected with *P. yoelii* NSM. 14 days later, the mice were sacrificed, and single-cell suspensions of mesenteric lymph node (MLN), lung, liver, spleen, and peripheral blood were prepared and counted. Then, different fluorescence labeled anti-CD3 and anti-γδTCR monoclonal antibodies were used to measure the frequency of γδT cells (**Figure 1D**). As shown in **Figure 1D**, the percentage of γδT cells in the infected mice spleen was significantly higher than that in the naive group (*P* < 0.01). The percentages of γδT cells in lung, liver, MLN and PBMC of infected mice were higher than those in naive mice (*P* < 0.05). Meanwhile, the absolute numbers of γδT cells in the spleen, MLN, lung, and liver significantly increased (*P* < 0.05) after malaria infection.

### Phenotypic and Functional Changes in Splenic γδT Cells in *P. yoelii* NSM Infected C57BL/6 Mice

**Figure 2:**
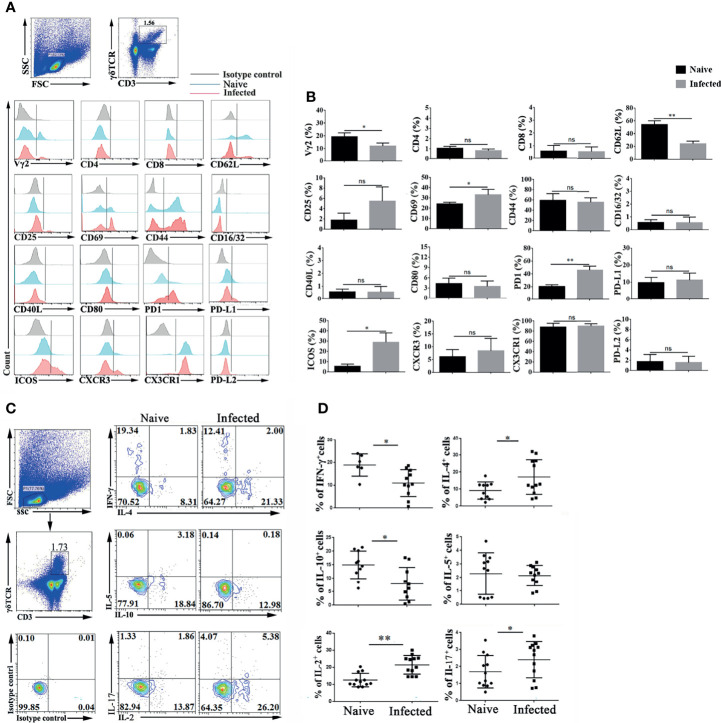
*Plasmodium* infection induced splenic γδT cell activation and differentiation. **(A, B)** Splenocytes were separated from naive and infected mice (14 days post infection) and then stained with monoclonal antibodies against mouse CD3, γδTCR, Vγ2, CD4, CD8, CD62 L, CD25, CD69, CD44, CD16/32, CD40 L, CD80, PD-1, PD-L1, PD-L2, ICOS, CXCR3 and CX3CR1 after cell surface staining. **(A)** A representative result of three independent experiments is shown. **(B)** The average expression of different surface molecules on γδT cells was calculated by FCM data. **(C, D)** Isolated splenocytes were stimulated by PMA and ionomycin, and the expression levels of cytokines (IFN-γ, IL-4, IL-5, IL-10, IL-2, and IL-17) were detected in CD3^+^ γδTCR^+^ cells after intracellular staining. **(C)** A representative of three independent experiments is shown. **(D)** The average expression levels of different cytokines on γδT cells were calculated by FCM data. The average of three independent experiments with 3-5 mice per group was shown and repeated three times with similar results. The error bars are SD, *P < 0.05, **P < 0.01. ns, no significance.

To explore the characteristics of γδT cells, the splenic single cell suspension was prepared, and T cell subpopulation (Vγ2, CD4, CD8 and CD44), activation or function (CD25, CD69, CD62L, CD40L, CD16/32, CD80, PD-1, PDL1and PDL2), and migration (CXCR3, CX3CR1, CXCR6 and CX3CR1) related molecules were detected by FCM (**Figure 2A**). As shown in **Figure 2B**, the proportion of Vγ2 expressing γδT cells significantly decreased after *P. yoelii* NSM infection (*P* < 0.01). The expression of the activation-associated molecule CD62L was significantly decreased (*P* < 0.01), while that of CD69 was increased (*P* < 0.05). The expression of function-related ICOS on γδT cells also increased (*P* < 0.05). Interestingly, the percentage of PD-1 expressing γδT cells was increased significantly (*P* < 0.01). Beyond that, the expression levels of CD44, CD16/32, CD40L, CD80, PD-L1, PD-L2, CXCR3, CX3CR1 and CX3CR1 were not significantly different between the naive group and the infected group (*P* > 0.05).

γδT cells can secrete multiple cytokines, such as IFN-γ, IL-4, IL-5, IL-6, IL-10, IL-13, IL-17 (Silva-Santos et al., 2019; Cha et al., 2020; Seifert et al., 2020). Spleen single cell suspensions were stimulated by PMA plus ionomycin, and intracellular cytokines were stained to examine cytokine production. As shown in **Figure 2C**, the expression of IFN-γ, IL-2, IL-4, IL-5, IL-10, and IL-17 was detected in γδT cells. The proportions of IFN-γ and IL-10 secreting γδT cells were decreased after *P. yoelii* NSM infection (*P <* 0.05) (**Figure 2D**). In contrast, the percentages of IL-2-, IL-4-, and IL-17-secreting γδT cells were increased in the infected group (*P <* 0.05). There was no significant difference in the secretion of IL-5 by γδT cells between naive and *P. yoelii* NSM infected mice (*P >* 0.05).

### *P. yoelii* NSM Infection Induces Transcriptomic Changes at the Single-Cell Level in Splenic γδT Cells

**Figure 3:**
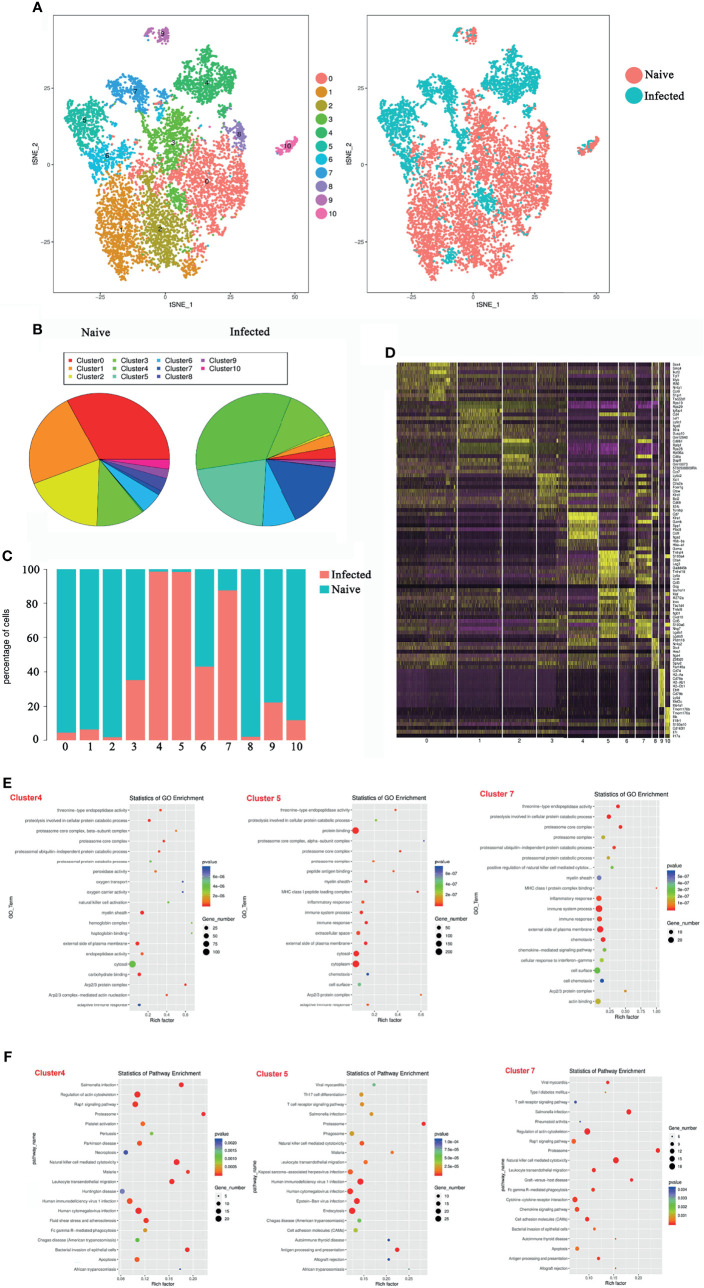
Single-cell transcriptomic analysis of splenic γδT cells from *P. yoelii* NSM infected mice. At 14 days after *P. yoelii* NSM infection, splenocytes were isolated from both naive and infected C57BL/6 mice. CD3^+^γδTCR^+^ cells were sorted using FACS, and the expression profile in single cells was detected using single-cell RNA sequencing (10× Genomics Chromium system). **(A)** t-distributed stochastic neighbour embedding visualization (t-SNE). All isolated γδT cells were classified into 11 clusters according to the properties of the RNA expression profile in each cell. Different colours represent different clusters, as indicated by the label *(left*). The cells from naive (red point) or infected (blue point) mice in each cluster are shown (*right*). **(B)** The percentage of cells in each cluster from naive (*left*) or infected (*right*) mice. **(C)** The ratio of each cluster in γδT cells from naive (blue) or infected (red) mice. **(D)** The heatmap of marker genes in each cell cluster. **(E)** The top 20 significant GO terms for the marker genes from 4, 5 and 7 cluster. The GO enrichment analysis revealed that the marker genes in each cluster were involved in innate and adaptive immunity. The left represents the GO term, the right represents enrichment, and the size of the solid circle indicates the number of genes. **(F)** The top 20 significant KEGG pathways for the marker genes from 4, 5 and 7 cluster. The KEGG pathway of marker genes in each cluster. The left represents the KEGG pathway, the right represents enrichment, and the size of the solid circle indicates the number of genes. A single experiment was performed for scRNA sequencing.

To explore the properties of splenic γδT cells in the progression of malaria infection, 14 days after *P. yoelii* NSM infection, CD3^+^γδTCR^+^ cells were sorted by FACS from splenocytes of both naive and infected C57BL/6 mice, and the RNA expression profile was determined using single-cell RNA sequencing (10× Genomics Chromium system). The CD3^+^γδTCR^+^ cells were gated firstly, the data were analyzed. As shown in **Figure 3A**, the isolated γδT cells were divided into 11 clusters by t-distributed stochastic neighbour embedding (t-SNE) visualization analysis. Detailed information on the marker genes in each cluster was shown in **Additional File 1: Table S1**. γδT cells from naive mice mainly contained clusters 0, 1, and 2, whereas γδT cells from infected mice mainly included clusters 4, 5, and 7 (**Figure 3B**). At the same time, the ratios of cells from naive or infected mice in each cluster were compared. More than 90% of γδT cells from naive mice were in clusters 0, 1, 2, and 8. In contrast, more than 90% of γδT cells from infected mice were in clusters 4 and 5 (**Figure 3C**). Moreover, the density of the marked genes in each cluster was listed and compared, as shown in a heatmap (**Figure 3D**).

To define the biological function of the marker genes from cluster 4, 5, and 7, GO and KEGG pathway enrichment was performed (**Figures 3E, F**). The marker genes in the infected group were mainly involved in the immune response. The GO enrichment analysis revealed that the marker genes in the infection group were involved in innate and adaptive immunity, such as “natural killer cell activation” and “adaptive immune response” in cluster 4 GO term enrichment; “MHC class I peptide loading complex”, “inflammatory response”, “immune system process”, “immune response”, and “adaptive immune response” in cluster 5 GO term enrichment; and “MHC class I peptide complex binding”, “inflammatory response”, “immune system process”, and “immune response” in cluster 7 GO term enrichment (**Figure 3E**).

Pathway enrichment analysis identified the marker genes in the infected group that were significantly enriched in “T cell receptor signalling pathway”, “Th17 cell differentiation”, “Natural killer cell-mediated cytotoxicity”, and some host defence against infectious disease processes (**Figure 3F**). These results suggested that *P. yoelii* NSM infection-induced γδT cells shared many key signalling molecules with other cells or against pathogen infection. This further demonstrated that γδT cells, especially in clusters 4, 5, and 7, are deeply involved in fighting *P. yoelii* NSM.

### The Effects of γδT Cells on the Infection Rate and Mortality of Malaria Parasites

**Figure 4:**
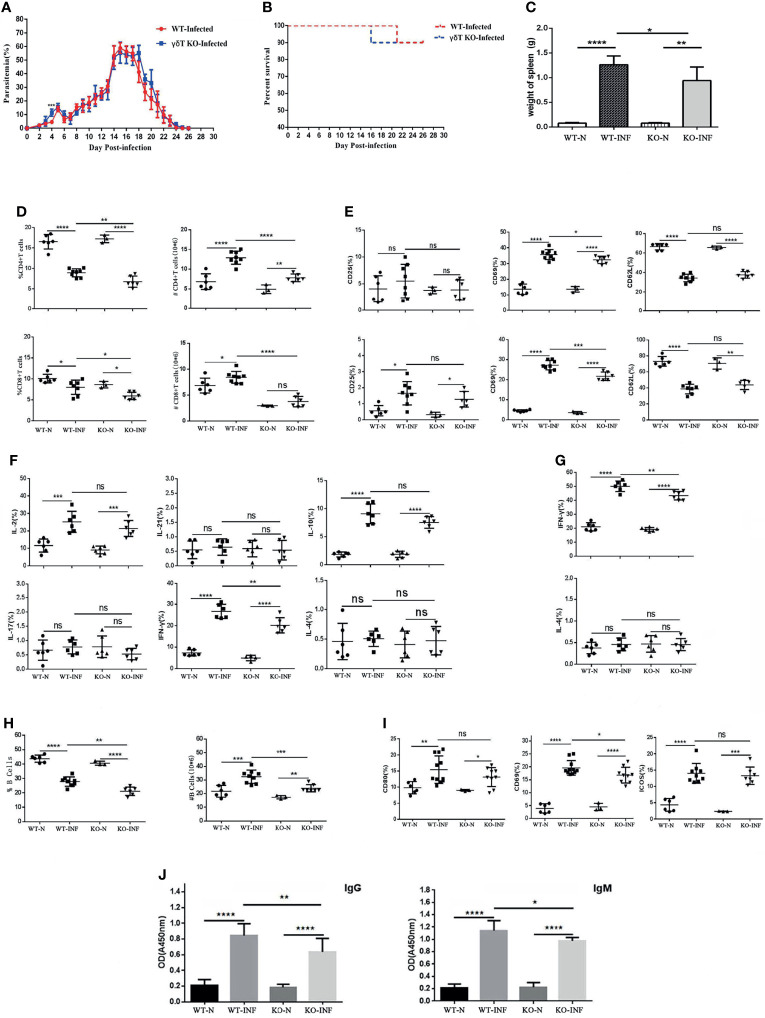
γδTCR KO decreased splenic T cell response and B cell response in *P. yoelii* NSM infected mice. Dynamic monitoring of percent parasitaemia **(A)** and percent survival **(B)** in wild-type C57BL/6 and γδTCR KO mice, which were both infected with *Plasmodium yoelii NSM*. *P<0.05. Representative results of two independent results are shown (N=10). **(C)** Mice were infected with *P. yoelii* NSM, and 14 days later, spleens were separated from both naive and *P. yoelii* NSM infected wild-type and KO mice. The weights of the spleens from each group of mice were measured (N=5-6). **(D)** FCM statistical graphs of the distribution and content of splenic CD4^+^ T cells and CD8^+^ T cells in the two infected and two naive groups. # refers to absolute number. **(E)** The expression levels of CD69, CD62L and CD25 on CD4^+^ T cells and CD8^+^ T cells in *P. yoelii* NSM infected mice compared with the two naive groups. **(F, G)** The cytokine expression levels of IFN-γ, IL-2, IL-4, IL-10, IL-17, and IL-21 in CD4^+^ T cells and CD8^+^ T cells obtained from *Plasmodium*-infected mice compared with the two naive groups. **(H)** The percentage and content of B cells in each group of mice were detected by FCM and counted. **(I)** The expression levels of CD69, ICOS and CD80 in B cells in each group of mice were detected by FCM. **(J)** Serum from both naive and infected C57BL/6 and γδTCR KO mice was collected and diluted 100 times. The contents of malaria-specific IgM and IgG antibodies were detected by ELISA. The OD values are shown. Representative results of three independent results are shown (N=5) and the error bars are SD. ****P < 0.0001, ***P < 0.001, **P < 0.01, *P < 0.05, ns, *P* > 0.05.

To record the percent parasitaemia and percent survival in C57BL/6 mice infected with *P. yoelii* NSM, female SPF 6–8 weeks old C57BL/6 mice and γδTCR KO mice were intraperitoneally infected with *P. yoelii* NSM (10^6^). Thin blood smears were obtained from blood of mice tails, stained with Giemsa, and counted daily from day 1 to day 26. As shown in **Figure 4A**, the level of parasitaemia in γδTCR KO mice was higher than that in wild-type mice on day 4 (*P* < 0.05). There was no significant difference in the survival rates of these two groups. Death of one γδT knockout mouse occurred on day 16, while one wild-type group died on day 21 (**Figure 4B**). 14 days later, the spleen was obtained and recorded weight. As shown in **Figure 4C**, the spleen weight in the infected mice was significantly higher than that in the uninfected control (*P* < 0.05), and the spleen weight in the infected γδTCR KO mice was lower than that in infected wild-type (WT) C57BL/6 mice (*P* < 0.05). In addition, blood was collected from these mice, the numbers of blood cells (WBCs, RBCs and PLTs) were counted. As shown in **Additional File 2: Figure S1**, the results indicated that in the blood of both infected wild-type and infected γδTCR KO mice, the number of WBCs was increased (*P* < 0.05) compared with that in the naive mice, while the numbers of RBCs and PLTs were significantly decreased (*P* < 0.01). Compared with infected WT mice, the number of WBCs was higher, and the numbers of RBCs and PLTs were significantly lower in the blood of infected γδTCR KO mice (*P* < 0.05).

To explore the effect of γδT cells on T cells, single-cell suspension from spleen in four groups, including WT-infected, γδTCR KO-infected, WT-naive, γδTCR KO-naive groups, were stained with fluorescence labelled CD3, CD4 and CD8. Based on **Figure 4D**, we found that the distribution of CD4^+^ T cells and CD8^+^ T cells decreased after *P. yoelii* NSM infection (*P* < 0.05). Interestingly, the γδTCR KO-infected group had lower distributions of CD4^+^ T cells and CD8^+^ T cells than the WT-infected group (*P* < 0.05). The absolute numbers of CD4^+^ T cells and CD8^+^ T cells were higher in the two infected groups (*P* < 0.05). The proportions of CD4^+^ T cells and CD8^+^ T cells in the WT-infected group were higher than those in the γδTCR KO infected group (*P* < 0.01).

As shown in **Figure 4E**, the expression levels of CD69 in the two infected groups were higher than those in two naive groups on both CD4^+^ and CD8^+^ cells (*P* < 0.01). The WT-infected group had more CD69 expression than the γδTCR KO infected group (*P*<0.05). This demonstrated that γδT cells could help activate both CD4 and CD8 T cells in the spleens of *P. yoelii* NSM infected mice. Although the expression of CD62L was significantly decreased in the two infected groups (*P* < 0.01), no significant difference was found between the two infected groups (*P* > 0.05).

To further explore the role of γδT cells in mediating the splenic T cell immune response, splenocytes were collected from these four groups of mice, stimulated with PMA and ionomycin, and intracellular cytokine staining was performed. As shown in **Figure 4F/4G**, CD4^+^ T cells were gated first, and the percentages of IL-2, IL-10, and IFN-γ expressing cells in the two infected groups were significantly increased compared with that in WT mice (*P* < 0.01). Notably, the percentage of IFN-γ expression cells in the splenic γδT cells from infected WT mice was higher than that from infected γδTCR KO mice (*P* < 0.01). The percentage of IFN-γ expressing CD8^+^ T cells in infected groups was higher than that in naive mice (*P* < 0.05), and a lower percentage of IFN-γ expressing cells was found in CD8^+^ cells from infected γδTCR KO mice (*P* < 0.05). The flow cytometry plots corresponding to these statistical graph results were in **Additional File 3: Figure S2**. These results demonstrated that γδT cells have significant promotion effects on T cell proliferation and activation after malaria infection, especially in promoting CD4^+^ T cells and CD8^+^ T cells to produce IFN-γ.

As shown in **Figure 4H**, the percentages of B cells in infected groups were lower than those in the naive groups (*P* < 0.01), and the percentage of B cells in the group of WT-infected mice was higher than that in γδTCR KO infected mice (*P* < 0.01). However, the absolute numbers of CD19^+^ B cells were significantly increased in the two infected groups compared with the two naive controls (*P* < 0.01). As expected, the number of B cells in the WT infected group was higher than that in the γδTCR KO-infected group (*P* < 0.01). The expression levels of CD80, ICOS and CD69 in B cells from infected groups were significantly higher than those in B cells from naive mice (*P* < 0.05, **Figure 4I**). Compared with infected γδTCR KO mice, a higher percentage of CD69 expressing B cells was found in the group of infected WT mice (*P* < 0.05). Moreover, blood was collected from these four groups of mice, serum was extracted and diluted, and the concentration of malaria specific antibodies was detected by ELISA. As shown in **Figure 4J**, the concentrations of malaria specific IgM and IgG were higher in the serum from malaria-infected mice *(P* < 0.01). Furthermore, the concentrations of malaria specific IgM and IgG were significantly decreased in the serum of the γδTCR KO-infected group, compared with the WT-infected group (*P* < 0.01). These results indicated that γδT cells could promote B cell activation, proliferation and antibody production.

## Discussion

γδT cells play an important role in resisting malaria infection in humans and mice (Pamplona and Silva-Santos, 2020). In this study, the properties of γδT cells were investigated in *P. yoelii* NSM infected C57BL/6 mice. The percentage and numbers of splenic γδT cells peaked at approximately days 12 to 16 after infection, which is the middle phase of *P. yoelii* NSM infection. In addition, we found that the frequency of γδT cells in the spleen was higher than in the liver, MLN, PBMC and lung of infected mice. This result indicated that γδT cells participate in the immune response in *P. yoelii* NSM infected mice as reported (Nadeem et al., 2019).

Vγ2 γδT cells is a sub-population of δT cells based on the type of the Vδ strain (Buus et al., 2016). It was reported that Vγ2 γδT cells could recruit neutrophils and deteriorate liver fibrosis induced by *Schistosoma japonicum* infection in C57BL/6 mice (Zheng et al., 2017). Another research indicated that Vγ2 cells expanded during malaria infection (Deroost and Langhorne, 2018). However, in our study the percentage of splenic Vγ2 γδT cells was decreased in *P. yoelii* NSM infected mice. It implied that other population of γδT cells might expand quickly in the spleen of *P. yoelii* NSM infected mice.

CD69 (Wang et al., 2014), inducible co-stimulatory molecule (ICOS) (Liu et al., 2021), programmed death 1 (PD-1) (Iwasaki et al., 2011) and L-selectin (CD62L) (Vassena et al., 2015) were T cells activation associated molecules. In our study, increased expression of CD69, ICOS, and PD-1, and the decreased expression of CD62L on the surface of infection induced splenic γδT cells demonstrating that *P. yoelii* NSM infection could induce γδT cell activation. It was reported that CD40L and ICOS can help T-B cell adhesion and antibody production by different manner in human (Liu et al., 2021).Significant higher percentage of ICOS and lower level of CD40L expressed on the splenic γδT cells from *P. yoelii* NSM infected mice, suggesting that ICOS might be an effective functional molecule for γδT cells in mediating immune responses during *P. yoelii* NSM infection. It was reported that γδT cells can express APC-related cell surface markers (Khan et al., 2014). However, we found that few of CD80, PD-L1, and PDL2 was expressed on γδT cells from both WT and infected mice. It implied that CD80, PD-L1, and PDL2 were not involved in γδT cells mediating *P. yoelii* NSM infection induced immune response in the spleen of C57BL/6 mice.

IFN-γ secreted by γδT cells is important for liver-stage *Plasmodium* infection (Buus et al., 2016), and the antibodies and IL-4-producing CD4^+^ T cell response play the major role during the chronic phase (Comeau et al., 2020). In our study, a higher percentage of IL-4, and a lower percentage of IFN-γ secreting γδT cells were found in the spleens of *P. yoelii* NSM infected mice. It implicated that γδT cells can exhibit Th2 conversion in this model. Together with the increased PD-1 expression, it seemed that functional exhaustion developed in *P. yoelii* NSM infection induced splenic γδT cells, as found in *Plasmodium vivax* exposed patients (Perez-Mazliah and Langhorne, 2014). IL-17 can recruit neutrophils, and induce inflammation (Gogoi et al., 2018). Significantly increased IL-17^+^ γδT cells was found in the spleens of *P. yoelii* NSM infected mice, which implied that γδT cells played roles in inducing inflammation in this model. IL-10 can inhibit the anti-malarial response mediated by T cells (Freitas Do Rosario and Langhorne, 2012). It was reported that non-Vγ9 γδT cells in the peripheral blood of patients with naturally acquired immunity against falciparum malaria had the potential to expand and produce IL-10 and IFN-γ (Taniguchi et al., 2017). Although, decreased of IL-10^+^ γδT cells was found in the spleen of *P. yoelii* NSM infected mice, it convinced that γδT possessed the ability to inhibit T cell immune response in the spleen of *P. yoelii* NSM infected mice.

Single-cell RNA sequencing (scRNA-seq) clustering helps elucidate cell-to-cell heterogeneity and uncover cell subgroups and cell dynamics at the group level (Peng et al., 2020). By single-cell RNA sequencing, *Pizzolato* et al. unveiled the shared and the distinct cytotoxic hallmarks of human TCRVγ1 and TCRVδ2 γδT lymphocytes (Pizzolato et al., 2019). scRNA-seq were performed to comprehensively catalog the heterogeneity of γδT cells derived from murine liver and thymus, and liver hematopoietic progenitor LSM cells were found able to differentiate into pre-γδT cells and functionally mature γδT cells (Hu et al., 2021). The sequencing data in this study provided great information for research in not only γδT cell differentiation, but also host anti-malaria process. The purified γδT cells from both naive and infected mice could be divided into 11 clusters. γδT cells from naive mice mainly contained clusters 0, 1, and 2, whereas γδT cells from infected mice mainly included clusters 4, 5, and 7. The GO and KEGG enrichment analysis furtherly revealed that the marker genes in clusters 4, 5, and 7 were involved in innate and adaptive immunity. These results not only demonstrated the diversity of γδT cells, but also indicated that γδT cells have differentiated and participated in host innate and adaptive anti-malaria immunity process. Similarly, γδT cells were reported to expand rapidly after resolution of acute parasitaemia, express specific cytokines, M-CSF, CCL5, CCL3, and were necessary for preventing parasitaemic recurrence (Mamedov et al., 2018). γδT cells expressing CD40L could promote dendritic cell activation and induced clearance of the *Plasmodium* parasites (Inoue et al., 2012). γδT cells were reported to be critical for the induction of sterile immunity during irradiated *Plasmodium* sporozoite vaccinations (Zaidi et al., 2017). Furthermore, the mechanism about γδT activation and differentiation induced by *Plasmodium* infection was unclear. 60 marker genes were identified for clusters 4, 5, and 7 in this study. These genes could provide useful information for exploring the mechanism of γδT activation and differentiation induced by *Plasmodium* infection in the future.

Next, γδTCR KO mice were infected by *P. yoelii* NSM to confirm the roles of γδT cells in the course of *P. yoelii* NSM infection. A significantly higher parasitaemia was found in γδTCR KO mice 4 days after infection (*P* < 0.05), suggesting that γδT cells played an important role in eliminating invasive *P. yoelii* NSM in the early stage of infection. Consisted with us, γδT cells have been shown to provide immune protection against blood-stage malaria in a granzyme and granulysin mediated innate immune mechanism (Hernandez-Castaneda et al., 2020). The level of parasitaemia in *P. yoelii* NSM infected WT and γδTCR KO mice were peaked at about 14 days after infection, no significant difference was found between these two groups. It may related to the expression of PD-1 on γδT cells. PD-1 is highly expressed in exhausted T cells and is associated with impaired effector function and increased apoptosis (He and Xu, 2020). It was reported that PD-1 signaling pathways could inhibit the cytotoxicity of human γδT cells (Hwang et al., 2021). Moreover, the weights of the spleens in the infected γδTCR KO mice were decreased implied that γδT cells might promote immune response in the spleen of *P. yoelii* NSM infected mice as found in *P. berghei* XAT infection (Inoue et al., 2013). However, increased number of WBCs and decreased numbers of RBCs were found in the blood of infected γδTCR KO mice. It might be related to that γδT cells can suppress *Plasmodium falciparum* blood-stage infection by forming immunological synapses with and lysis iRBCs, and destroying the parasite in patients (Junqueira et al., 2021).

The T cell-mediated immune response plays a key role during malaria. CD4^+^ Th cells can modulate the type of immune response (Kumar et al., 2020; Soon et al., 2020), and CD8^+^ Tc cells are essential for the clearance of intracellular pathogens and serve as targets for malaria vaccine research (Holz et al., 2020). It has long been observed that γδT cells can act as antigen-presenting cells (APCs), which are a bridge between innative and adaptive immune responses (Tyler et al., 2017). Here, a lower percentage of CD69 and a higher percentage of CD62L were expressed on CD4^+^ and CD8^+^ T cells, decreased percentages of IL-2, IL-10, and IFN-γ expressing CD4^+^ Th cells, and lower percentages of IFN-γ expressing CD8^+^ T cells were found in the spleens of *Plasmodium* infected γδTCR KO mice than that in the infected WT mice (*P* < 0.05). It suggested that γδT cells could promote splenic T cell response in the course of *P. yoelii* NSM infection.

Moreover, the humoral immune response also plays an important role in preventing clinical malaria (Joyner et al., 2019). Decreased numbers of B cells in the spleen, decreased expression of CD69 on B cells, and lower levels of IgG and IgM antibodies in the serum were found in *P. yoelii* NSM infected γδTCR KO mice compared with infected WT mice. It indicated that γδT cells could enhance the humoral immune response against *Plasmodium* infection. Consistent with our results, γδT cells were found modulating size and productivity of preimmune peripheral B cell populations (Huang et al., 2016).

## Conclusions

Overall, our study suggested that the expansion of γδT cells in cluster 4, 5 and 7 could enhance both cellular and humoral immune responses in the spleen of *Plasmodium yoelii nigeriensis* NSM infected C57BL/6 mice.

## Data Availability Statement

The datasets presented in this study can be found in online repositories. The names of the repository/repositories and accession number(s) can be found below: bioproject/, PRJNA702594 and PRJNA702837, https://www.st-va.ncbi.nlm.nih.gov/.

## Ethics Statement

The animal study was reviewed and approved by The Institutional Animal Care and Use Committee of Guangzhou Medical University (2015–012).

## Author Contributions

XW and JH conceived the study. HX, SX, and MW performed the *in vitro* cellular test. AX and JL performed histological experiment. QY, HW, CF, and FS analysed the results. HH and ZY prepared parasite and animal. XW and JH contributed to the writing of the paper. All authors read and approved the final manuscript.

## Funding

This research was supported by grants from the Natural Science Foundation of China (81771696), the Guangdong Provincial Education Department (2016KZDXM033), the Natural Science Foundation of Guangdong Province (2020A1515010251, 2021A1515011032), Guangzhou Science and Technology Project (202002030082), Scientific Research Project of Traditional Chinese Medicine Bureau of Guangdong Province (20212127) and the Open Foundation Key Laboratory of Tropical Diseases Control (Sun Yat-sen University), Ministry of Education (2021kfkt03).

## Conflict of Interest

The authors declare that the research was conducted in the absence of any commercial or financial relationships that could be construed as a potential conflict of interest.

## Publisher’s Note

All claims expressed in this article are solely those of the authors and do not necessarily represent those of their affiliated organizations, or those of the publisher, the editors and the reviewers. Any product that may be evaluated in this article, or claim that may be made by its manufacturer, is not guaranteed or endorsed by the publisher.

## Supplementary Material

Additional file 1: Table S1 Information on the marker genes in each cluster.

Additional file 2: Figure S1
**(A–C)** At 14 days after *P. yoelii* NSM infection, blood samples from each group of mice were collected. The numbers of WBCs, RBCs, and PLTs in the blood were measured. Representative results of three independent results are shown (N=5) and the error bars are SD. *****P* < 0.0001, ****P* < 0.001, ***P* < 0.01, **P* < 0.05.

Additional file 3: Figure S2 The supplementary data for flow cytometry. **(A)** The FCM plots of distribution and content of CD4^+^ T cells and CD8^+^ T cells in the two infected and two naive groups. **(B, C)** The FCM plots of expression levels of activated markers on CD4^+^ T cells and CD8^+^ T cells in *P. yoelii* NSM infected mice compared with the two naive groups. **(D, E)** The FCM plots of expression levels of cytokines on CD4^+^ T cells and CD8^+^ T cells. **(F)** The FCM plots of percentage and content of B cells in each group of mice. **(G)** The FCM plots of expression levels of activated markers on B cells in *P. yoelii* NSM infected mice compared with the two naive groups.

## References

[B1] Abel A. Steeg C. Aminkiah F. Addai-Mensah O. Addo M. Gagliani N.. (2018). Differential Expression Pattern of Co-Inhibitory Molecules on CD4(+) T Cells in Uncomplicated Versus Complicated Malaria. Sci. Rep. 8, 4789. doi: 10.1038/s41598-018-22659-1 29555909 PMC5859076

[B2] Akbari M. Kimura K. Bayarsaikhan G. Kimura D. Miyakoda M. Juriasingani S.. (2018). Nonspecific CD8(+) T Cells and Dendritic Cells/Macrophages Participate in Formation of CD8(+) T Cell-Mediated Clusters Against Malaria Liver-Stage Infection. Infect. Immun. 86 (4), e00717–17. doi: 10.1128/IAI.00717-17 PMC5865025 29426043

[B3] Aye R. Sutton H. J. Nduati E. W. Kai O. Mwacharo J. Musyoki J.. (2020). Malaria Exposure Drives Both Cognate and Bystander Human B Cells to Adopt an Atypical Phenotype. Eur. J. Immunol. 50, 1187–1194. doi: 10.1002/eji.201948473 32222961 PMC7611263

[B4] Bigorra L. Larriba I. Gutierrez-Gallego R. (2019). Machine Learning Algorithms for the Detection of Spurious White Blood Cell Differentials Due to Erythrocyte Lysis Resistance. J. Clin. Pathol. 72, 431–437. doi: 10.1136/jclinpath-2019-205820 30992342

[B5] Born W. K. Kemal A. M. O'Brien R. L. (2013). Diversity of Gammadelta T-Cell Antigens. Cell Mol. Immunol. 10, 13–20. doi: 10.1038/cmi.2012.45 23085946 PMC4003174

[B6] Buus T. B. Geisler C. Lauritsen J. P. (2016). The Major Diversification of Vgamma1.1(+) and Vgamma2(+) Thymocytes in Mice Occurs After Commitment to the Gammadelta T-Cell Lineage. Eur. J. Immunol. 46, 2363–2375. doi: 10.1002/eji.201646407 27418188

[B7] Caccamo N. La Mendola C. Orlando V. Meraviglia S. Todaro M. Stassi G.. (2011). Differentiation, Phenotype, and Function of Interleukin-17-Producing Human Vgamma9Vdelta2 T Cells. Blood 118, 129–138. doi: 10.1182/blood-2011-01-331298 21505189

[B8] Carding S. R. Egan P. J. (2002). Gammadelta T Cells: Functional Plasticity and Heterogeneity. Nat. Rev. Immunol. 2, 336–345. doi: 10.1038/nri797 12033739

[B9] Chaves Y. O. Da C. A. Pereira M. L. de Lacerda M. V. Coelho-Dos-Reis J. G. Martins-Filho O. A.. (2016). Immune Response Pattern in Recurrent Plasmodium Vivax Malaria. Malar. J. 15, 445. doi: 10.1186/s12936-016-1501-5 27581163 PMC5007810

[B10] Cha H. Xie H. Jin C. Feng Y. Xie S. Xie A.. (2020). Adjustments of Gammadelta T Cells in the Lung of Schistosoma Japonicum-Infected C56BL/6 Mice. Front. Immunol. 11, 1045. doi: 10.3389/fimmu.2020.01045 32582168 PMC7287124

[B11] Comeau K. Paradis P. Schiffrin E. L. (2020). Human and Murine Memory Gammadelta T Cells: Evidence for Acquired Immune Memory in Bacterial and Viral Infections and Autoimmunity. Cell Immunol. 357, 104217. doi: 10.1016/j.cellimm.2020.104217 32979762 PMC9533841

[B12] Dantzler K. W. Jagannathan P. (2018). Gammadelta T Cells in Antimalarial Immunity: New Insights Into Their Diverse Functions in Protection and Tolerance. Front. Immunol. 9, 2445. doi: 10.3389/fimmu.2018.02445 30405634 PMC6206268

[B13] Deroost K. Langhorne J. (2018). Gamma/Delta T Cells and Their Role in Protection Against Malaria. Front. Immunol. 9. doi: 10.3389/fimmu.2018.02973 PMC6306408 30619330

[B14] Dutta S. Tewari A. Balaji C. Verma R. Moitra A. Yadav M.. (2018). Strain-Transcending Neutralization of Malaria Parasite by Antibodies Against Plasmodium Falciparum Enolase. Malar. J. 17, 304. doi: 10.1186/s12936-018-2455-6 30126436 PMC6102825

[B15] Elizalde-Torrent A. Trejo-Soto C. Mendez-Mora L. Nicolau M. Ezama O. Gualdron-Lopez M.. (2021). Pitting of Malaria Parasites in Microfluidic Devices Mimicking Spleen Interendothelial Slits. Sci. Rep. 11, 22099. doi: 10.1038/s41598-021-01568-w 34764379 PMC8585870

[B16] Freitas Do Rosario A. P. Langhorne J. (2012). T Cell-Derived IL-10 and its Impact on the Regulation of Host Responses During Malaria. Int. J. Parasitol. 42, 549–555. doi: 10.1016/j.ijpara.2012.03.010 22549022

[B17] Gogoi D. Biswas D. Borkakoty B. Mahanta J. (2018). Exposure to Plasmodium Vivax is Associated With the Increased Expression of Exhaustion Markers on Gammadelta T Lymphocytes. Parasite Immunol. 40, e12594. doi: 10.1111/pim.12594 30276843

[B18] Hartwig T. Pantelyushin S. Croxford A. L. Kulig P. Becher B. (2015). Dermal IL-17-Producing Gammadelta T Cells Establish Long-Lived Memory in the Skin. Eur. J. Immunol. 45, 3022–3033. doi: 10.1002/eji.201545883 26332438

[B19] Hernandez-Castaneda M. A. Happ K. Cattalani F. Wallimann A. Blanchard M. Fellay I.. (2020). Gammadelta T Cells Kill Plasmodium Falciparum in a Granzyme- and Granulysin-Dependent Mechanism During the Late Blood Stage. J. Immunol. 204, 1798–1809. doi: 10.4049/jimmunol.1900725 32066596 PMC7086388

[B20] He X. Xu C. (2020). PD-1: A Driver or Passenger of T Cell Exhaustion? Mol. Cell 77, 930–931. doi: 10.1016/j.molcel 32142689

[B21] Hirunpetcharat C. Good M. F. (1998). Deletion of Plasmodium Berghei-Specific CD4^+^ T Cells Adoptively Transferred Into Recipient Mice After Challenge With Homologous Parasite. Proc. Natl. Acad. Sci. U. S. A. 95, 1715–1720. doi: 10.1073/pnas.95.4.1715 9465082 PMC19161

[B22] Holz L. E. Chua Y. C. de Menezes M. N. Anderson R. J. Draper S. L. Compton B. J.. (2020). Glycolipid-Peptide Vaccination Induces Liver-Resident Memory CD8(+) T Cells That Protect Against Rodent Malaria. Sci. Immunol. 5 (48), eaaz8035. doi: 10.1126/sciimmunol.aaz8035 32591409

[B23] Hovav A. H. Wilharm A. Barel O. Prinz I. (2020). Development and Function of gammadeltaT Cells in the Oral Mucosa. J. Dent. Res. 99, 498–505. doi: 10.1177/0022034520908839 32091949

[B24] Huang Y. Getahun A. Heiser R. A. Detanico T. O. Aviszus K. Kirchenbaum G. A.. (2016). Gammadelta T Cells Shape Preimmune Peripheral B Cell Populations. J. Immunol. 196, 217–231. doi: 10.4049/jimmunol.1501064 26582947 PMC4684964

[B25] Huang X. Huang S. Ong L. C. Lim J. C. Hurst R. J. Mushunje A. T.. (2016). Differential Spleen Remodeling Associated With Different Levels of Parasite Virulence Controls Disease Outcome in Malaria Parasite Infections. Msphere 1 (1), e00018–15. doi: 10.1128/mSphere.00018-15 PMC4863626 27303680

[B26] Hu Y. Fang K. Wang Y. Lu N. Sun H. Zhang C. (2021). Single-Cell Analysis Reveals the Origins and Intrahepatic Development of Liver-Resident IFN-Gamma-Producing Gammadelta T Cells. Cell Mol. Immunol. 18, 954–968. doi: 10.1038/s41423-021-00656-1 33692482 PMC8115257

[B27] Hviid L. Kurtzhals J. A. Adabayeri V. Loizon S. Kemp K. Goka B. Q.. (2001). Perturbation and Proinflammatory Type Activation of V Delta 1(+) Gamma Delta T Cells in African Children With Plasmodium Falciparum Malaria. Infect. Immun. 69, 3190–3196. doi: 10.1128/IAI.69.5.3190-3196.2001 11292740 PMC98276

[B28] Hwang H. J. Lee J. J. Kang S. H. Suh J. K. Choi E. S. Jang S.. (2021). The BTLA and PD-1 Signaling Pathways Independently Regulate the Proliferation and Cytotoxicity of Human Peripheral Blood γδ T Cells. Immun. Inflamm. Dis. 9, 274–287. doi: 10.1002/iid3.390 33332777 PMC7860523

[B29] Inoue S. Niikura M. Mineo S. Kobayashi F. (2013). Roles of IFN-Gamma and Gammadelta T Cells in Protective Immunity Against Blood-Stage Malaria. Front. Immunol. 4, 258. doi: 10.3389/fimmu.2013.00258 24009610 PMC3756480

[B30] Inoue S. Niikura M. Takeo S. Mineo S. Kawakami Y. Uchida A.. (2012). Enhancement of Dendritic Cell Activation *via* CD40 Ligand-Expressing γδ T Cells is Responsible for Protective Immunity Toplasmodium Parasites. Proc. Natl. Acad. Sci. 109, 12129–12134. doi: 10.1073/pnas.1204480109 22778420 PMC3409789

[B31] Iwasaki M. Tanaka Y. Kobayashi H. Murata-Hirai K. Miyabe H. Sugie T.. (2011). Expression and Function of PD-1 in Human Gammadelta T Cells That Recognize Phosphoantigens. Eur. J. Immunol. 41, 345–355. doi: 10.1002/eji.201040959 21268005

[B32] Joyner C. J. Brito C. Saney C. L. Joice C. R. Smith M. L. Lapp S. A.. (2019). Humoral Immunity Prevents Clinical Malaria During Plasmodium Relapses Without Eliminating Gametocytes. PLoS Pathog. 15, e1007974. doi: 10.1371/journal.ppat.1007974 31536608 PMC6752766

[B33] Junqueira C. Polidoro R. B. Castro G. Absalon S. Liang Z. Sen S. S.. (2021). Gammadelta T Cells Suppress Plasmodium Falciparum Blood-Stage Infection by Direct Killing and Phagocytosis. Nat. Immunol. 22, 347–357. doi: 10.1038/s41590-020-00847-4 33432229 PMC7906917

[B34] Keswani T. Sarkar S. Sengupta A. Bhattacharyya A. (2016). Role of TGF-Beta and IL-6 in Dendritic Cells, Treg and Th17 Mediated Immune Response During Experimental Cerebral Malaria. Cytokine 88, 154–166. doi: 10.1016/j.cyto.2016.08.034 27632786

[B35] Khan M. W. Curbishley S. M. Chen H. C. Thomas A. D. Pircher H. Mavilio D.. (2014). Expanded Human Blood-Derived gammadeltaT Cells Display Potent Antigen-Presentation Functions. Front. Immunol. 5, 344. doi: 10.3389/fimmu.2014.00344 25101086 PMC4107971

[B36] Kumarasingha R. Ioannidis L. J. Abeysekera W. Studniberg S. Wijesurendra D. Mazhari R.. (2020). Transcriptional Memory-Like Imprints and Enhanced Functional Activity in Gammadelta T Cells Following Resolution of Malaria Infection. Front. Immunol. 11, 582358. doi: 10.3389/fimmu.2020.582358 33154754 PMC7591758

[B37] Kumar R. Loughland J. R. Ng S. S. Boyle M. J. Engwerda C. R. (2020). The Regulation of CD4(+) T Cells During Malaria. Immunol. Rev. 293, 70–87. doi: 10.1111/imr.12804 31674682

[B38] Langhorne J. Holder A. A. (1998). “αβ and γδt Cells in the Immune Response to the Erythrocytic Stages of Malaria in Mice,” in Encyclopedia of Immunology, 2nd ed. Delves P. J. (Oxford: Elsevier), 1658–1663.

[B39] Levine J. H. Simonds E. F. Bendall S. C. Davis K. L. Amir E. D. Tadmor M. D.. (2015). Data-Driven Phenotypic Dissection of AML Reveals Progenitor-Like Cells That Correlate With Prognosis. Cell 162, 184–197. doi: 10.1016/j.cell.2015.05.047 26095251 PMC4508757

[B40] Libera J. Wittner M. Kantowski M. Woost R. Eberhard J. M. de Heer J.. (2020). Decreased Frequency of Intestinal CD39(+) Gammadelta(+) T Cells With Tissue-Resident Memory Phenotype in Inflammatory Bowel Disease. Front. Immunol. 11, 567472. doi: 10.3389/fimmu.2020.567472 33072107 PMC7541837

[B41] Li J. Cai B. Qi Y. Zhao W. Liu J. Xu R.. (2016). UTR Introns, Antisense RNA and Differentially Spliced Transcripts Between Plasmodium Yoelii Subspecies. Malar. J. 15, 30. doi: 10.1186/s12936-015-1081-9 26791272 PMC4721144

[B42] Liu Z. Liu S. Zhang Y. Zeng W. Wang S. Ji P.. (2021). Distinct Roles of ICOS and CD40L in Human T-B Cell Adhesion and Antibody Production. Cell Immunol. 368, 104420. doi: 10.1016/j.cellimm.2021.104420 34418679

[B43] Lopez C. Yepes-Perez Y. Hincapie-Escobar N. Diaz-Arevalo D. Patarroyo M. A. (2017). What Is Known About the Immune Response Induced by Plasmodium Vivax Malaria Vaccine Candidates? Front. Immunol. 8, 126. doi: 10.3389/fimmu.2017.00126 28243235 PMC5304258

[B44] Mamedov M. R. Scholzen A. Nair R. V. Cumnock K. Kenkel J. A. Oliveira J. H. M.. (2018). A Macrophage Colony-Stimulating-Factor-Producing γδ T Cell Subset Prevents Malarial Parasitemic Recurrence. Immunity 48, 350–363. doi: 10.1016/j.immuni.2018.01.009 29426701 PMC5956914

[B45] Nadeem A. Ahmad S. F. Al-Harbi N. O. Al-Harbi M. M. Ibrahim K. E. Kundu S.. (2019). Inhibition of Spleen Tyrosine Kinase Signaling Protects Against Acute Lung Injury Through Blockade of NADPH Oxidase and IL-17A in Neutrophils and Gammadelta T Cells Respectively in Mice. Int. Immunopharmacol. 68, 39–47. doi: 10.1016/j.intimp.2018.12.062 30611000

[B46] Nielsen M. M. Witherden D. A. Havran W. L. (2017). Gammadelta T Cells in Homeostasis and Host Defence of Epithelial Barrier Tissues. Nat. Rev. Immunol. 17, 733–745. doi: 10.1038/nri.2017.101 28920588 PMC5771804

[B47] Ortiz-Ruiz A. Postigo M. Gil-Casanova S. Cuadrado D. Bautista J. M. Rubio J. M.. (2018). Plasmodium Species Differentiation by non-Expert on-Line Volunteers for Remote Malaria Field Diagnosis. Malar. J. 17, 54. doi: 10.1186/s12936-018-2194-8 29378588 PMC5789591

[B48] Pamplona A. Silva-Santos B. (2020). Gammadelta T Cells in Malaria: A Double-Edged Sword. FEBS J. 288 (4), 1118–1129. doi: 10.1111/febs.15494 32710527 PMC7983992

[B49] Peng L. Tian X. Tian G. Xu J. Huang X. Weng Y.. (2020). Single-Cell RNA-Seq Clustering: Datasets, Models, and Algorithms. RNA Biol. 17 (6), 765–783. doi: 10.1080/15476286.2020.1728961 32116127 PMC7549635

[B50] Perez-Mazliah D. Langhorne J. (2014). CD4 T-Cell Subsets in Malaria: TH1/TH2 Revisited. Front. Immunol. 5, 671. doi: 10.3389/fimmu.2014.00671 25628621 PMC4290673

[B51] Pizzolato G. Kaminski H. Tosolini M. Franchini D. M. Pont F. Martins F.. (2019). Single-Cell RNA Sequencing Unveils the Shared and the Distinct Cytotoxic Hallmarks of Human TCRVdelta1 and TCRVdelta2 Gammadelta T Lymphocytes. Proc. Natl. Acad. Sci. U. S. A. 116, 11906–11915. doi: 10.1073/pnas.1818488116 31118283 PMC6576116

[B52] Saavedra-Langer R. Marapara J. Valle-Campos A. Durand S. Vasquez-Chasnamote M. E. Silva H.. (2018). IgG Subclass Responses to Excreted-Secreted Antigens of Plasmodium Falciparum in A Low-Transmission Malaria Area of the Peruvian Amazon. Malar. J. 17, 328. doi: 10.1186/s12936-018-2471-6 30200987 PMC6131892

[B53] See P. Lum J. Chen J. Ginhoux F. (2018). A Single-Cell Sequencing Guide for Immunologists. Front. Immunol. 9, 2425. doi: 10.3389/fimmu.2018.02425 30405621 PMC6205970

[B54] Seifert A. M. List J. Heiduk M. Decker R. von Renesse J. Meinecke A. C.. (2020). Gamma-Delta T Cells Stimulate IL-6 Production by Pancreatic Stellate Cells in Pancreatic Ductal Adenocarcinoma. J. Cancer Res. Clin. Oncol. 146, 3233–3240. doi: 10.1007/s00432-020-03367-8 32865617 PMC7679341

[B55] Silva-Santos B. Mensurado S. Coffelt S. B. (2019). Gammadelta T Cells: Pleiotropic Immune Effectors With Therapeutic Potential in Cancer. Nat. Rev. Cancer 19, 392–404. doi: 10.1038/s41568-019-0153-5 31209264 PMC7614706

[B56] Soon M. Lee H. J. Engel J. A. Straube J. Thomas B. S. Pernold C.. (2020). Transcriptome Dynamics of CD4(+) T Cells During Malaria Maps Gradual Transit From Effector to Memory. Nat. Immunol. 21, 1597–1610. doi: 10.1038/s41590-020-0800-8 33046889

[B57] Subelj L. Bajec M. (2011). Unfolding Communities in Large Complex Networks: Combining Defensive and Offensive Label Propagation for Core Extraction. Phys. Rev. E Stat. Nonlin. Soft. Matter. Phys. 83, 36103. doi: 10.1103/PhysRevE.83.036103 21517554

[B58] Sundling C. Ronnberg C. Yman V. Asghar M. Jahnmatz P. Lakshmikanth T.. (2019). B Cell Profiling in Malaria Reveals Expansion and Remodelling of CD11c+ B Cell Subsets. JCI Insight 19 (7), 392–404. doi: 10.1172/jci.insight.126492 PMC6538354 30939125

[B59] Sun G. Yang S. Cao G. Wang Q. Hao J. Wen Q.. (2018). Gammadelta T Cells Provide the Early Source of IFN-Gamma to Aggravate Lesions in Spinal Cord Injury. J. Exp. Med. 215, 521–535. doi: 10.1084/jem.20170686 29282251 PMC5789408

[B60] Taniguchi T. Md M. K. Nonaka D. Toma H. Li C. Narita M.. (2017). A Unique Subset of Gammadelta T Cells Expands and Produces IL-10 in Patients With Naturally Acquired Immunity Against Falciparum Malaria. Front. Microbiol. 8, 1288. doi: 10.3389/fmicb.2017.01288 28769886 PMC5515829

[B61] Tyler C. J. McCarthy N. E. Lindsay J. O. Stagg A. J. Moser B. Eberl M. (2017). Antigen-Presenting Human Gammadelta T Cells Promote Intestinal CD4(+) T Cell Expression of IL-22 and Mucosal Release of Calprotectin. J. Immunol. 198, 3417–3425. doi: 10.4049/jimmunol.1700003 28330898 PMC5392732

[B62] Vassena L. Giuliani E. Koppensteiner H. Bolduan S. Schindler M. Doria M. (2015). HIV-1 Nef and Vpu Interfere With L-Selectin (CD62L) Cell Surface Expression To Inhibit Adhesion and Signaling in Infected CD4+ T Lymphocytes. J. Virol. 89, 5687–5700. doi: 10.1128/JVI.00611-15 25822027 PMC4442509

[B63] Victor J. R. Lezmi G. Leite-de-Moraes M. (2020). New Insights Into Asthma Inflammation: Focus on iNKT, MAIT, and gammadeltaT Cells. Clin. Rev. Allergy Immunol. 59, 371–381. doi: 10.1007/s12016-020-08784-8 32246390

[B64] Wang K. Hou Y. Wang X. Han G. (2014). Expression Kinetics of CD69 Molecule by CD3(+) Lymphocytes and gammadeltaT Cells Under Three Different Activating Modalities. Zhonghua Xue Ye Xue Za Zhi 35, 753–754. doi: 10.3760/cma.j.issn.0253-2727.2014.08.020 25152129

[B65] Wei H. Jin C. Peng A. Xie H. Xie S. Feng Y.. (2021). Characterization of gammadeltaT Cells in Lung of Plasmodium Yoelii-Infected C57BL/6 Mice. Malar. J. 20, 89. doi: 10.1186/s12936-021-03619-z 33588839 PMC7885449

[B66] White N. J. (2017). Malaria Parasite Clearance. Malar. J. 16, 88. doi: 10.1186/s12936-017-1731-1 28231817 PMC5324257

[B67] Wipasa J. Xu H. Stowers A. Good M. F. (2001). Apoptotic Deletion of Th Cells Specific for the 19-kDa Carboxyl-Terminal Fragment of Merozoite Surface Protein 1 During Malaria Infection. J. Immunol. 167, 3903. doi: 10.4049/jimmunol.167.7.3903 11564808

[B68] Wu D. Wu P. Qiu F. Wei Q. Huang J. (2017). Human gammadeltaT-Cell Subsets and Their Involvement in Tumor Immunity. Cell Mol. Immunol. 14, 245–253. doi: 10.1038/cmi.2016.55 27890919 PMC5360884

[B69] Xu C. Su Z. (2015). Identification of Cell Types From Single-Cell Transcriptomes Using a Novel Clustering Method. Bioinformatics 31, 1974–1980. doi: 10.1093/bioinformatics/btv088 25805722 PMC6280782

[B70] Xu H. Wipasa J. Yan H. Zeng M. Makobongo M. O. Finkelman F. D.. (2002). The Mechanism and Significance of Deletion of Parasite-Specific CD4(+) T Cells in Malaria Infection. J. Exp. Med. 195, 881–892. doi: 10.1084/jem.20011174 11927632 PMC2193727

[B71] Yang J. Y. Wang F. Zhou G. (2021). Characterization and Function of Circulating Mucosal-Associated Invariant T Cells and gammadeltaT Cells in Oral Lichen Planus. J. Oral. Pathol. Med. 5 (5), e126492. doi: 10.1111/jop.13250 34637577

[B72] Zaidi I. Diallo H. Conteh S. Robbins Y. Kolasny J. Orr-Gonzalez S.. (2017). γδ T Cells Are Required for the Induction of Sterile Immunity During Irradiated Sporozoite Vaccinations. J. Immunol. 199, 3781–3788. doi: 10.4049/jimmunol.1700314 29079696 PMC5698172

[B73] Zheng L. Hu Y. Wang Y. Huang X. Xu Y. Shen Y.. (2017). Recruitment of Neutrophils Mediated by Vgamma2 Gammadelta T Cells Deteriorates Liver Fibrosis Induced by Schistosoma Japonicum Infection in C57BL/6 Mice. Infect. Immun. 85 (8), e01020–16. doi: 10.1128/IAI.01020-16 PMC5520426 28507072

[B74] Zhou Q. H. Wu F. T. Pang L. T. Zhang T. B. Chen Z. (2020). Role of gammadeltaT Cells in Liver Diseases and its Relationship With Intestinal Microbiota. World J. Gastroenterol. 26, 2559–2569. doi: 10.3748/wjg.v26.i20.2559 32523311 PMC7265152

