# Supplementary figures and images for: Properties and Roles of γδT Cells in Plasmodium yoelii nigeriensis NSM Infected C57BL/6 Mice
Source: Front Cell Infect Microbiol. 2022 Jan 20;11:788546. doi: 10.3389/fcimb.2021.788546 (PMC8811364; doi:10.3389/fcimb.2021.788546)

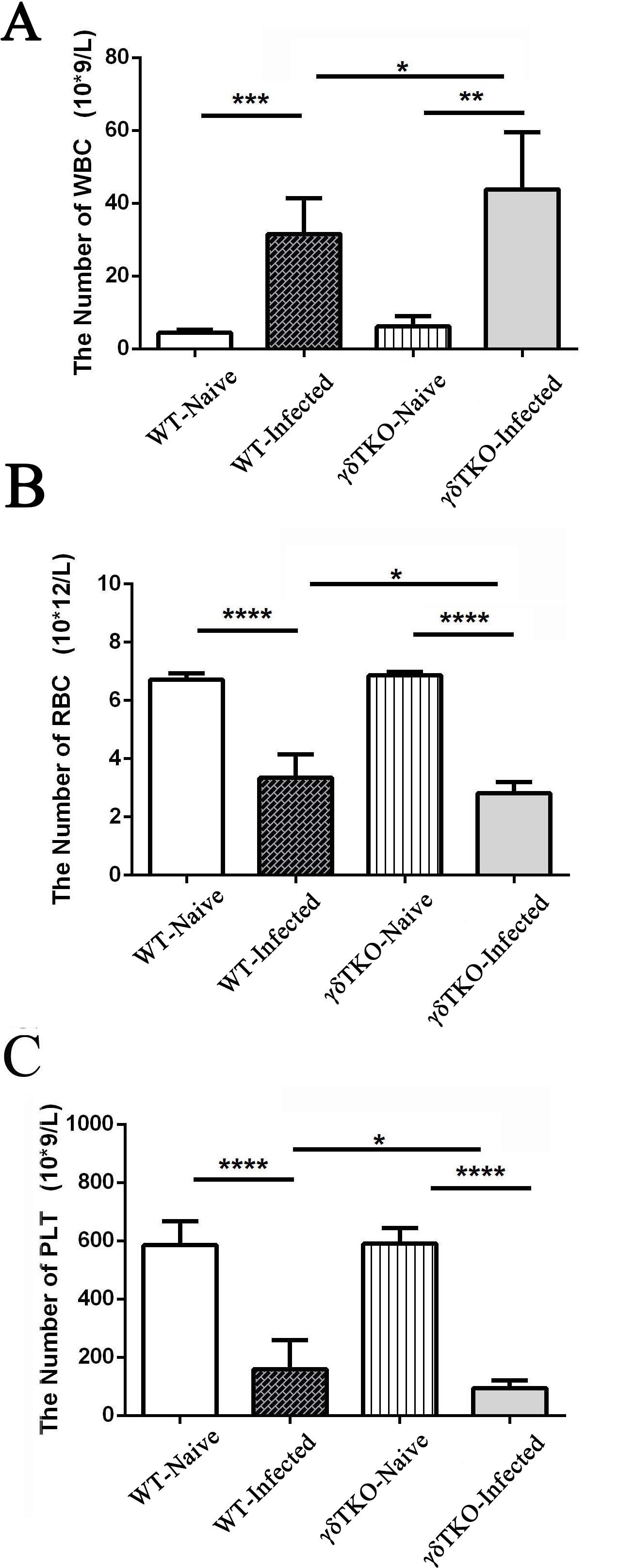

Supplement: Additional file 2: Figure S1 — (A–C) At 14 days after P. yoelii NSM infection, blood samples from each group of mice were collected. The numbers of WBCs, RBCs, and PLTs in the blood were measured. Representative results of three independent results are shown (N=5) and the error bars are SD. ****P < 0.0001, ***P < 0.001, **P < 0.01, *P < 0.05. [file Image_1.jpeg]

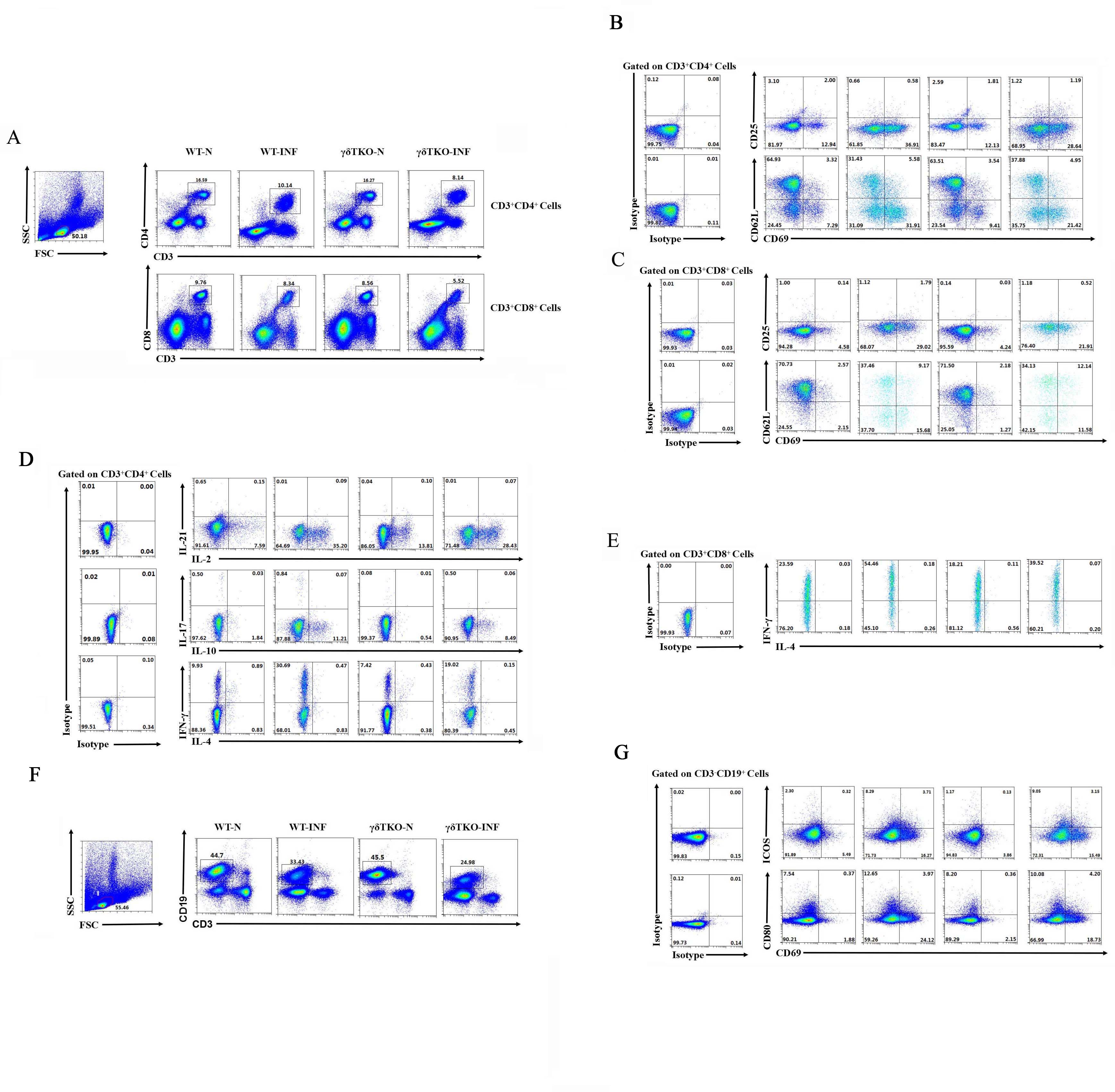

Supplement: Additional file 3: Figure S2 — The supplementary data for flow cytometry. (A) The FCM plots of distribution and content of CD4+ T cells and CD8+ T cells in the two infected and two naive groups. (B, C) The FCM plots of expression levels of activated markers on CD4+ T cells and CD8+ T cells in P. yoelii NSM infected mice compared with the two naive groups. (D, E) The FCM plots of expression levels of cytokines on CD4+ T cells and CD8+ T cells. (F) The FCM plots of percentage and content of B cells in each group of mice. (G) The FCM plots of expression levels of activated markers on B cells in P. yoelii NSM infected mice compared with the two naive groups. [file Image_2.jpeg]
